# Editorial: Emerging talents in human neuroscience: neuromodulation 2023

**DOI:** 10.3389/fnhum.2023.1354465

**Published:** 2024-01-05

**Authors:** Vincenzo Di Lazzaro

**Affiliations:** ^1^Department of Medicine and Surgery, Unit of Neurology, Neurophysiology, Neurobiology and Psychiatry, Università Campus Bio-Medico di Roma, Roma, Italy; ^2^Fondazione Policlinico Universitario Campus Bio-Medico, Roma, Italy

**Keywords:** neuromodulation, transcranial magnetic stimulation, transcranial direct current stimulation, non-invasive brain stimulation, TMS-EEG

In recent decades, there has been a growing interest in non-invasive neuromodulation. Several non-invasive brain stimulation (NIBS) techniques have been introduced to target and modulate cerebral cortex circuits in the attempt to restore normal function in several neuropsychiatric disorders (Lefaucheur et al., 2020). Beyond its potential therapeutic effects, neuromodulation also allows a functional evaluation of several brain networks contributing to a better understanding of human brain physiology.

Several NIBS techniques are already FDA-cleared for specific indications such as depression (Denison and Morrell, 2022) and the application of NIBS appears very promising for an increasing number of disorders. Thus, NIBS has an exciting future and is emerging as a neurology subspecialty and an excellent career opportunity for young clinicians and researchers (Peruzzotti-Jametti et al., 2013). The aim of the present Research Topic was to offer young talents interested in NIBS the opportunity to be the lead authors of innovative contributions to the field.

Cruciani et al. explore the contribution of TMS-EEG in the understanding of the pathophysiology of neurological disorders. The combination of transcranial magnetic stimulation with electroencephalography (TMS-EEG) is a cutting-edge technique that, despite some methodological challenges, allows the assessment of cortical function unbiased by spinal excitability. This approach holds promise but calls for standardization to ensure reliability.

The methodological heterogeneity of neurophysiological studies is a frequent criticism against neuromodulation. In this regard, Osnabruegge et al. underscore the importance of guidelines and standardized methodologies for enhancing the reliability and accuracy of neurophysiological measurements, especially in TMS research. To reduce variability, Agboada et al. propose an optimized semi-automated motor hotspot search technique to be used in TMS research. This method, when compared to manual hotspot research, has proven to be highly accurate and user-friendly (Agboada et al.).

The need for standardized methods is the topic of the article by Kanig et al.. They present a systematic review and meta-analysis focusing on the test-retest reliability of 15 studies employing various rTMS techniques on the primary motor cortex of healthy individuals. Their findings reveal small to moderate reliability in the identified studies, with no favored protocol based on aftereffect sizes. This work emphasizes the need for higher reliability to establish rTMS as an effective clinical treatment.

NIBS techniques are known to induce neuroplastic changesthat outlast the period of stimulation – offering the chance to treat conditions in which cortical excitability is altered, such as dementias. Norata et al. present a comprehensive review on the use of transcranial direct current stimulation (tDCS) for treating semantic variant primary progressive aphasia. The authors investigate the effectiveness of several tDCS protocols and stimulation sites, concluding that, despite the heterogeneity of the employed methodologies, tDCS has almost consistently shown efficacy in enhancing patients' performance in semantic tasks. However, the caveat is always the same, since the application of various NIBS protocols needs standardization for a better diffusion of neuromodulation as a reliable treatment option.

Lastly, Wimmer et al. present a nice contribution to this Research Topic, exploring the innovative realm of non-invasive neuromodulation through neurofeedback (NF). Their study on individuals with binge-eating disorder demonstrates the potential of NF-based treatments, highlighting specific strategies for modulating food-related responses. This study enhances our understanding of NF techniques and provides insights for future targeted interventions.

In conclusion, this Research Topic encapsulates diverse research efforts addressing critical aspects of neuromodulation techniques. The emerging talents who led the studies carefully navigated the evolving landscape of NIBS, emphasizing the need for methodological consistency and standardization. Additionally, they highlighted the significance of exploring innovative approaches to fully unlock the potential of these techniques in clinical applications.

As these emerging leaders explore new pathways, their contributions not only advance scientific understanding but also hold the potential for treating neurological disorders. The synergy of youthful curiosity and cutting-edge technologies promises a future where neuromodulation continues to thrive as a dynamic field, offering hope and solutions for some of the most complex challenges in medicine.

## Author contributions

VD: Writing – original draft.
